# Efficacy and Satisfaction Evaluation of Rapid Rehabilitation Nursing Intervention in Patients with Laparoscopic Myomectomy

**DOI:** 10.1155/2022/9412050

**Published:** 2022-07-05

**Authors:** Rong Song, Caifeng Chen, Li Shang

**Affiliations:** ^1^Department of Obstetrics and Gynecology, Anhui Wannan Rehabilitation Hospital (The Fifth People's Hospital of Wuhu), Wuhu, China; ^2^Shiyan Maternal and Child Health Hospital, Shiyan, China

## Abstract

**Objective:**

To evaluate the efficacy and satisfaction of rapid rehabilitation nursing in patients with laparoscopic myomectomy.

**Methods:**

In this retrospective study, a total of 60 patients scheduled for a laparoscopic myomectomy in our hospital from January 2019 to February 2022 were enrolled and assigned at a ratio of 1 : 1 to receive either routine nursing (routine group) or rapid rehabilitation nursing (study group) according to different nursing methods. Outcome measures included nursing efficiency and nursing satisfaction.

**Results:**

Rapid rehabilitation nursing resulted in less intraoperative blood loss, shorter postoperative exhaust time and hospital stay, and a lower incidence of bladder irritation (123.56 ± 30.68, 22.54 ± 5.07, 5.70 ± 1.06, and 4.55%) versus the routine nursing (185.78 ± 32.78, 31.02 ± 5.28, 7.57 ± 2.19, 18.18%) (*P* < 0.05). Rapid rehabilitation nursing was associated with a lower incidence of complications (3.33%) versus routine nursing (20.00%) (*P* < 0.05). The eligible patients receiving rapid rehabilitation nursing showed a significantly higher satisfaction (96.67%) versus routine nursing (80.00%) (*P* < 0.05).

**Conclusion:**

Rapid rehabilitation nursing is effective in the nursing of patients after laparoscopic myomectomy by minimizing the physical and mental stress of patients, shortening the length of hospital stay, reducing the occurrence of related complications, and boosting the quality of life of patients, so it is worthy of clinical application.

## 1. Introduction

Uterine fibroids [1] are benign tumors due to the proliferation of uterine smooth muscle tissue and remain one of the most common tumors in the female population [2]. At present, the incidence of clinical uterine fibroids has been on a gradual rise. Although the pathogenesis of uterine fibroids remains unknown, risk factors such as heredity, hormones, stem cells, cell mutations in the normal myometrium, sex hormones, and local growth factors have been identified. The disease exhibits a high prevalence among women aged 30-50 [3] but is less frequently seen in those under 20. Conservative therapy may be considered for asymptomatic patients. The symptoms of uterine fibroids are associated with the growth location, rate of growth, degeneration, and complications of fibroids but are independent of the size and number of fibroids. Symptoms in patients with multiple subserosal fibroids could be insidious, while they may cause irregular vaginal bleeding or menorrhagia, leading to dysmenorrhea, increased menstrual flow, miscarriage, and infertility [4, 5]. Surgical treatment is the mainstay of treatment for uterine fibroids [6], including myomectomy and hysterectomy that can be performed either through the abdomen or the vagina, and laparoscopic myomectomy is considered the most common and effective [7, 8]. Notwithstanding its effectiveness, the operation is traumatic and results in stress response, complications, and adverse reactions, compromising the prognosis of the patient [9]. Therefore, it necessitates fine nursing care to consolidate the treatment results. Rapid rehabilitation nursing [10] refers to the use of perioperative validated objective methods through the cooperation of various departments such as surgery, anesthesia, nursing, and nutrition, to mitigate patients' perioperative stress, minimize the risk of intraoperative complications and adverse reactions, and lower the risk of death [11, 12]. Nonetheless, there is a lack of research that analyzes the effect of rapid rehabilitation nursing in patients with laparoscopic myomectomy. With the advancement of medical technology, the demand for better medical services has been increasing. Rapid rehabilitation surgical nursing is a supplement and improvement of the traditional nursing concept, which can effectively alleviate and control the physical and mental stress of surgical patients and minimize their physical and mental damage, with maximum benefits and the best quality of service, and has received wide recognition in clinical practice. To fill the gap, the present study was conducted to shed light on clinical practice.

## 2. Materials and Methods

### 2.1. Study Population

In this retrospective study, a total of 60 patients scheduled for a laparoscopic myomectomy in our hospital from January 2019 to February 2022 were enrolled and assigned at a ratio of 1 : 1 to a routine group or a study group according to different nursing methods. The patients in the routine group were 28-54 years, with an average age of 45.97 ± 5.98 years, 29 cases were married, and 1 cases were unmarried. The patients in the study group were 26-52 years, with an average age of 42.67 ± 6.44 years, 28 cases were married, and 2 cases were unmarried. The general data were comparable between the two groups. This study was approved by the ethics committee of the Fifth People's Hospital of Wuhu, (Approval number: [2020]10) .

### 2.2. Inclusion and Exclusion Criteria

Inclusion criteria were as follows: (a) all met the diagnostic criteria for uterine fibroids and met the surgical indications; (2) the patients had complete clinical data; (3) the patients and their families were informed of the study and voluntarily signed the consent form; (4) the patients had a diagnosis of benign uterine and ovarian diseases (ovarian cysts, uterine fibroids, etc.) confirmed by comprehensive clinical manifestations, imaging findings, and pathological findings; and (5) the patients had no history of previous abdominal surgical treatment.

Exclusion criteria were as follows: (1) with vital organ lesions; (2) with contraindications to surgery; (3) with unconsciousness or cognitive impairment; (4) with malignant tumors; (5) with chronic diseases such as hypertension, diabetes, coronary heart disease, and chronic obstructive pulmonary disease; (6) with severe liver and kidney organ dysfunction; (7) with intestinal obstruction and diarrhea; and (8) with low compliance and unable to cooperate with treatment and nursing intervention.

### 2.3. Nursing Methods

The routine group received routine nursing, including examination guidance, preoperative precautions, health education, and psychological care. Polyethylene Glycol-Electrolyte Powder (2 boxes) (Jiangxi Hengkang Pharmaceutical Co. Ltd., SFDA Approval No.H20020031) and warm water (2000mL) followed by the insertion of the urinary catheter and the monitoring of vital signs. After the operation, the patients were given basic nursing and rehabilitation nursing, and the patient's condition and the occurrence of complications were observed and recorded.

The study group adopted rapid rehabilitation nursing. (1) Psychological nursing. Psychological nursing included psychological status assessment, targeted counseling, and comfort, active communication, and mitigation of negative emotions. (2) Health education. Health education was provided for the patients to improve their awareness of the disease conditions, and the precautions related to laparoscopic myomectomy were given to allow patients and their families to understand the entire process of the onset, treatment, and recovery of the disease and improve their compliance with treatment. (3) Preoperative care. The patients were instructed to fast from solid food for 6 hours and liquid food for 4 hours before surgery to keep the intestines clean, and turpentine and alcohol were used to clean the patient's umbilicus. (4) Intraoperative nursing. The nursing staff provided sufficient comfort and guidance to the patients to relieve their physiological stress, and the temperature and humidity of the operating room were appropriately adjusted. (5) Postoperative care. The changes in the patient's vital signs were closely monitored, the catheter was maintained unobstructed, and the patients were aided to turn over. The nursing staff massaged the back of the patients to reduce pain and swelling, and reasonable early activities 6 hours after the operation were encouraged to promote blood circulation and reduce the occurrence of complications. The patient's postoperative recovery was assessed, and they were instructed to perform rehabilitation training. The puncture sites and the perineum were kept dry and clean to avoid retrograde infection.

### 2.4. Evaluation Criteria

1. The clinical indicators of the two groups of patients, including intraoperative blood loss, postoperative exhaust time, the incidence of bladder irritation, and hospitalization time, were recorded and analyzed
2. The occurrence of postoperative complications in the two groups was recorded, including postoperative bleeding, infection, and frequent urination, and the total incidence of each group was calculated
3. The self-developed *Nursing Satisfaction Questionnaire* was used to rate the satisfaction of nursing, with a reliability validity of 0.798 and retest reliability of 0.801. 88 questionnaires were distributed, and 88 valid questionnaires were actually received, with a 100% recovery rate. Patients scored according to their satisfaction with the nursing, with each question scoring 5 points. A score of <70 points indicates dissatisfied, a score of 70-89 points indicates satisfied, and a score of ≥90 points indicates highly satisfied. Satisfaction = (highly satisfied + satisfied)/total number of cases × 100%.

### 2.5. Statistical Analysis

SPSS22.0 software was used for data analyses. Enumeration data (*n* (%)) and measurement data (*x* ± s) were analyzed using the chi-square and *t*-test, respectively. Differences were considered statistically significant at *P* < 0.05.

## 3. Results

### 3.1. Clinical Indicators

Rapid rehabilitation nursing resulted in less intraoperative blood loss, shorter postoperative exhaust time and hospital stay, and a lower incidence of bladder irritation (123.56 ± 30.68, 22.54 ± 5.07, 5.70 ± 1.06, and 4.55%) in the study group versus the routine nursing (185.78 ± 32.78, 31.02 ± 5.28, 7.57 ± 2.19, 18.18%) (*P* < 0.05). See Table 1.

### 3.2. Complications

There were 2 (6.67%) cases of postoperative bleeding, 1 (3.33%) cases of infection, and 3 (10.00%) cases of frequent urination in the routine group, while there were 0 (0.00%) case of postoperative bleeding, 0 (0.00%) case of infection, and 1 (3.33%) case of frequent urination in the study group. Rapid rehabilitation nursing was associated with a lower incidence of complications (3.33%) versus routine nursing (20.00%) (*P* < 0.05). See Table 2.

### 3.3. Satisfaction

In the routine group, 10 (33.33%) cases were highly satisfied, 14 (46.67%) cases were satisfied, and 6 (20.00%) cases were dissatisfied. In the study group, 14 (46.67%) cases were highly satisfied, 15 (50.00%) cases were satisfied, and 1 (3.33%) case was dissatisfied. The eligible patients receiving rapid rehabilitation nursing showed a significantly higher satisfaction (96.67%) versus routine nursing (80.00%) (*P* < 0.05). See Table 3. Rapid rehabilitation nursing resulted in less intraoperative blood loss, shorter postoperative exhaust time and hospital stay, and a lower incidence of bladder irritation (123.56 ± 30.68, 22.54 ± 5.07, 5.70 ± 1.06, and 4.55%) versus the routine nursing (185.78 ± 32.78, 31.02 ± 5.28, 7.57 ± 2.19, and 18.18%) (*P* < 0.05). Rapid rehabilitation nursing was associated with a lower incidence of complications (3.33%) versus routine nursing (20.00%) (*P* < 0.05). The eligible patients receiving rapid rehabilitation nursing showed a significantly higher satisfaction (96.67%) versus routine nursing (80.00%) (*P* < 0.05).

## 4. Discussion

Currently, laparoscopic surgery is the mainstay to treat uterine fibroids [13], with the merits of small trauma and rapid postoperative recovery [14, 15]. However, the intraoperative stress response results in a high incidence of postoperative complications, considerably compromising the postoperative rehabilitation [16, 17]. Therefore, effective nursing intervention is essential to promote the postoperative recovery of patients following laparoscopic myomectomy. The concept of rapid rehabilitation nursing is a new nursing concept [18] to control perioperative pathophysiological changes through various modalities and improve the postoperative condition of patients [19]], to reduce the physical and mental harm caused by surgery.

Previous research has shown that [20] the prognosis of patients after laparoscopic myomectomy can be positively improved with good nursing, with a robust speed of recovery. The results of the present study showed that rapid rehabilitation nursing resulted in less 5intraoperative blood loss, shorter postoperative exhaust time and hospital stay, and a lower incidence of bladder irritation and complications versus routine nursing. This nursing mode relieves the psychological burden of the patient through preoperative psychological care, thereby reducing the intraoperative stress response; moreover, preoperative diet control ensures the nutritional needs of patients, improves immunity, and reduces the stress response caused by malnutrition. During surgery, patients are given thermal insulation care to ensure smooth operation and avoid multiple complications caused by low temperature. In addition, the patient's postoperative vital signs are monitored, and targeted pain intervention is carried out according to the degree of pain. The lower limbs of the patient are massaged to further reduce the occurrence of postoperative complications. All these measures actively accelerate the postoperative recovery and inhibit the adverse stress response of the body to improve the patient's clinical indicators.

Here, the total satisfaction of patients in the study group was significantly higher than that in the routine group. The possible reason is that rapid rehabilitation nursing lessens the surgery-related abnormal reactions, effectively promotes the rapid recovery of patients' functions, significantly reduces the comprehensive complications of patients after surgery, and realizes the scientific allocation of medical and health resources. Rapid rehabilitation nursing strengthens communication with patients during the operation and the related treatment of postoperative complications, so as to ease and calm the tension and anxiety of the patients, which enables better cooperation of patients and minimizes the occurrence of side effects, enhances the quality of life of patients, and accelerates the recovery of patients. Although the present study sheds light on the robustness of rapid rehabilitation nursing on patients undergoing laparoscopic myomectomy, certain limitations merit attention. For instance, this study only used routine care as a control, which is not widely covered and might bias the results towards null. Hence, future studies involving more nursing modalities as contrasts are warranted. Rapid rehabilitation care could improve the postoperative indexes due to the significantly shorter preoperative fasting time, the absence of mechanical bowel cleansing preparation, and the earlier time of out-of-bed activity, all of which were conducive to promoting gastrointestinal motility and facilitating the recovery of gastrointestinal function. The synergistic effect of all factors eventually effectively reduced the impact of surgery on the normal function of the patient's gastrointestinal tract and enhanced the patient's early recovery. Intraoperative control of the patient's body temperature and operating room temperature effectively lowers the incidence of intraoperative complications such as bleeding and infection due to hypothermia in patients.

Rapid rehabilitation nursing is effective in the nursing of patients after laparoscopic myomectomy by minimizing the physical and mental stress of patients, shortening the length of hospital stay, reducing the occurrence of related complications, and boosting the quality of life of patients, so it is worthy of clinical application. The limitations of this study are the absence of a randomized multicenter study and the inclusion bias in the retrospective study, which will be included in future studies to obtain additional follow-up data.

## Figures and Tables

**Table 1:** Comparison of clinical indicators between the two groups before and after nursing intervention ($\bar{x}\pm s$, %).

Groups	*n*	Intraoperative blood loss (ml)	Postoperative exhaust time (h)	Incidence of bladder irritation	Hospital stay (d)
Routine group	30	185.78 ± 32.78	31.02 ± 5.28	8 (18.18)	7.57 ± 2.19
Study group	30	123.56 ± 30.68	22.54 ± 5.07	2 (4.55)	5.70 ± 1.06
*t*/*x*^2^	—	9.192	7.684	4.602	4.388
*P*	—	<0.001	<0.001	0.044	<0.001

**Table 2:** Comparison of the incidence of complications between the two groups before and after nursing intervention (%).

Groups	*n*	Postoperative bleeding	Infection	Frequent urination	Incidence
Routine group	30	2 (6.67)	1 (3.33)	3 (10.00)	6 (20.00)
Study group	30	0 (0.00)	0 (0.00)	1 (3.33)	1 (3.33)
*x* ^2^	—	4.043			
*P*	—	0.044			

**Table 3:** Comparison of satisfaction scores of two groups of patients before and after nursing intervention (%).

Groups	*n*	Highly satisfied	Satisfied	Dissatisfied	Total
Routine group	30	10 (33.33)	14 (46.67)	6 (20.00)	24 (80.00)
Study group	30	14 (46.67)	15 (50.00)	1 (3.33)	29 (96.97)
*x* ^2^	—	4.043			
*P*	—	0.044			

## Data Availability

The datasets used during the present study are available from the corresponding author upon reasonable request.
